# Toxic Effects of Indoxyl Sulfate on Osteoclastogenesis and Osteoblastogenesis

**DOI:** 10.3390/ijms222011265

**Published:** 2021-10-19

**Authors:** Jia-Fwu Shyu, Wen-Chih Liu, Cai-Mei Zheng, Te-Chao Fang, Yi-Chou Hou, Chiz-Tzung Chang, Ting-Ying Liao, Yin-Cheng Chen, Kuo-Cheng Lu

**Affiliations:** 1 Department of Biology and Anatomy, National Defense Medical Center, Taipei 110, Taiwan; shyujeff@mail.ndmctsgh.edu.tw (J.-F.S.); waynliu55@gmail.com (W.-C.L.); 2 Division of Nephrology, Department of Internal Medicine, Taipei Hospital, Ministry of Health and Welfare, New Taipei City 242, Taiwan; tingying.liao8@gmail.com (T.-Y.L.); ian.ycchen@gmail.com (Y.-C.C.); 3 Division of Nephrology, Department of Internal Medicine, Taipei Medical University Hospital, Taipei Medical University, Taipei 110, Taiwan; fangtechao@gmail.com; 4 Division of Nephrology, Department of Internal Medicine, Taipei Medical University Shuang Ho Hospital, New Taipei City 235, Taiwan; 11044@s.tmu.edu.tw; 5 Graduate Institute of Clinical Medicine, College of Medicine, Taipei Medical University, Taipei 110, Taiwan; 6 Division of Nephrology, Department of Internal Medicine, School of Medicine, College of Medicine, Taipei Medical University, Taipei 110, Taiwan; 7 Division of Nephrology, Department of Medicine, Cardinal-Tien Hospital, School of Medicine, Fu-Jen Catholic University, New Taipei City 234, Taiwan; athletics910@gmail.com; 8 College of Medicine, China Medical University, Taichung City 406, Taiwan; ma273737@gmail.com; 9 Division of Nephrology, Department of Medicine, Taipei Tzu Chi Hospital, Buddhist Tzu Chi Medical Foundation, New Taipei City 231, Taiwan

**Keywords:** aryl hydrocarbon receptor, bone remodeling, indoxyl sulfate, osteoblast, osteoclast

## Abstract

Uremic toxins, such as indoxyl sulfate (IS) and kynurenine, accumulate in the blood in the event of kidney failure and contribute to further bone damage. To maintain the homeostasis of the skeletal system, bone remodeling is a persistent process of bone formation and bone resorption that depends on a dynamic balance of osteoblasts and osteoclasts. The aryl hydrocarbon receptor (AhR) is a ligand-activated transcription factor that regulates the toxic effects of uremic toxins. IS is an endogenous AhR ligand and is metabolized from tryptophan. In osteoclastogenesis, IS affects the expression of the osteoclast precursor nuclear factor of activated T cells, cytoplasmic 1 (NFATc1) through AhR signaling. It is possible to increase osteoclast differentiation with short-term and low-dose IS exposure and to decrease differentiation with long-term and/or high-dose IS exposure. Coincidentally, during osteoblastogenesis, through the AhR signaling pathway, IS inhibits the phosphorylation of ERK, and p38 reduces the expression of the transcription factor 2 (Runx2), disturbing osteoblastogenesis. The AhR antagonist resveratrol has a protective effect on the IS/AhR pathway. Therefore, it is necessary to understand the multifaceted role of AhR in CKD, as knowledge of these transcription signals could provide a safe and effective method to prevent and treat CKD mineral bone disease.

## 1. Introduction

Chronic kidney disease (CKD) causes CKD-MBD, which is a broad inclusion of abnormalities in systemic bone mineral metabolism and the cardiovascular system. The disease comprises characteristics of laboratory abnormalities, vascular/soft tissue calcification, and bone metabolism deterioration [1]. These disorders of the skeleton are the predominant causes of bone loss and fractures in patients with CKD. In the case of CKD, the incidence of fractures [2], morbidity, and mortality are higher than in the general population [3], even among patients with adequate control of calcium, phosphate, and parathyroid hormone (PTH) levels with calcimimetics [4,5]. This suggests that, beyond calcitriol, PTH, calcium, and phosphate, uremic toxins could play an important role in the modulation of bone metabolism in CKD. Among uremic toxins, such as kynurenine (KYN) and indoxyl sulfate (IS), the metabolites of tryptophan are elevated in uremic patients compared with healthy population, and they may increase oxidative stress and induce inflammatory processes and cell damage [6,7]. A growing number of studies indicate that kynurenine build-up in CKD increases Reactive Oxygen Species (ROS) generation and oxdiative distress to suppress the proliferation and differentiation of osteoblasts and increase the risk of bone fracture [8,9,10]. In another way, IS affects bone formation through decreasing the differentiation of osteoclasts and promoting osteoblast apoptosis, thus patients often develop adynamic or low-turnover bone disease in the early stages of CKD [7]. Numerous studies have acknowledged that the coincidence of dysregulated bone demineralisation and increased vascular calcification provides a close relationship between CKD, bone disorders, and vascular diseases [11,12,13]. IS was heavily discussed recently in the literature regarding the degenerating bone quantity and quality, which can be described as “uremic osteoporosis” [14]. This new concept of CKD-related bone fragility highlights the complicated relationship between bone quality, fractures, and mortality in CKD patients [15]. In addition, at the beginning of CKD, the production of calcitriol is reduced, and the synthesis of the Wnt pathway inhibitors (DKK1, SOST) is increased, leading to the accumulation of uremic toxins [16]. Therefore, the elevated levels of uremic toxins disrupt bone remodeling with altered osteocaclastogenesis and osteoblastogenesis, both of which intensify the severity of CKD-MBD. 

Bone remodeling is correlated with bone resorption and bone formation to maintain bone homeostasis. The rate of remodeling is influenced by several factors, such as PTH, sex steroids, and uremic toxins [17]. IS is proven to be associated with altered bone remodeling abnormalities [17,18,19]. Intriguingly, IS induces an excessive toxicity in CKD by acting as an endogenous ligand of the aryl hydrocarbon receptor (AhR) [20,21]. However, still little is known about the molecular mechanisms of IS on renal bone disease in patients with CKD.

AhR has been recognized as a receptor of environmental pollutants and a mediator of chemical toxicity for the last 30 years. AhR, as a ligand-activated transcription factor, is involved in different cellular courses, including cellular development, neural signals, the functioning of the epithelial barrier, and the response to xenobiotics and antioxidants [22]. In the genomic pathway, AhR binds to a ligand and translocates into the nucleus, thereby regulating gene expression under the control of exogenous response components. In the non-genomic pathway, the ligand binds to AhR and activates the inflammatory response and other transcription factors, such as nuclear factor-kappa B (NF-ƙB) and activator protein 1 (AP-1) [23]. Recent studies have paid more attention to the effects of AhR on the immune system and bone remodeling [24,25,26], and the role of AhR during CKD has attracted more scientific interest. Although the analysis of AhR is becoming increasingly extensive, a deeper understanding is still needed to clarify the importance of AhR in the bone remodeling of CKD–BMD. Therefore, in this review, we focus on the impact of AhR on bone remodeling under the influence of IS in the scenario of CKD and its related signal pathway, therefore hoping to provide a theoretical basis on how to improve bone remodeling in CKD patients.

## 2. Osteogenesis and CKD

### 2.1. Normal Bone Remodeling Cycle

**Figure 1:**
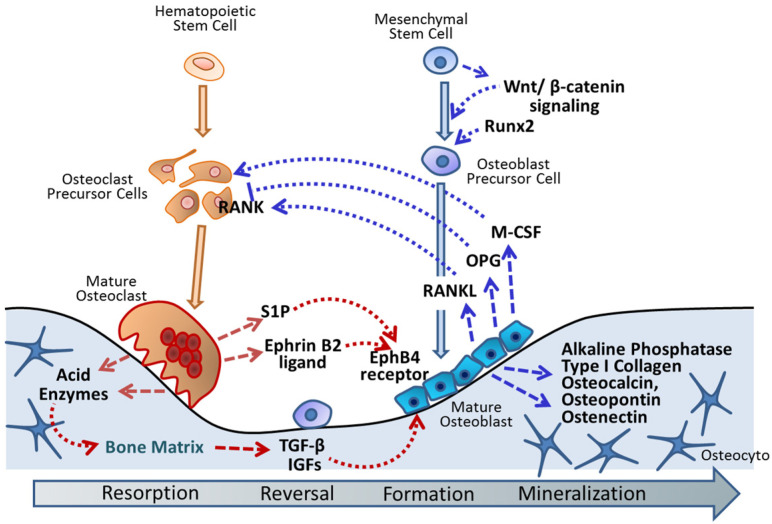
The bone remodeling cycle. The purpose of constant bone remodeling is to maintain normal bone mass as a dynamic equilibrium between resorption and formation. Mesenchymal progenitors trigger the activation of canonical Wnt/β-catenin signaling to promote the differentiation of osteoblasts. Osteoblasts produce runt-related transcription factor 2 (Runx2) as the earliest marker and various extracellular matrix proteins, such as ALP, type I collagen, osteocalcin, osteopontin, and ostenectin, to accomplish bone formation and mineralization. Mature osteoblasts produce RANKL to achieve osteoclast differentiation. Alternatively, osteoblasts also release OPG to attenuate the osteoclast stimulant pathway. Macrophage colony stimulation factor (M-CSF) is another modulator of osteoclast differentiation produced by osteoblasts. Osteoclasts secrete acid and enzymes to absorb the bone matrix and release transforming growth factor-beta (TGF-β) and insulin-like growth factors (IGFs) to control the activity of osteoblasts. Moreover, osteoclasts express ephrin B2 ligand and sphingosine 1-phosphate (S1P) to bind with the EphB4 receptor on osteoblasts, which can promote osteoblast differentiation and can suppress osteoclast differentiation. Thus, there is a close link between osteoclastic bone resorption and osteoblastic bone formation.

Bone remodeling occurs in the basic multicellular unit (BMU) [27], which contains the osteoclasts, osteoblasts, and osteocytes within the bone remodeling cavity (Figure 1). Bone remodeling is a lifelong and persistent process of bone resorption and formation that is initiated by osteoclasts absorbing old bone, followed by mononuclear cell reversal, osteoblast formation, osteoblast mineralization, and the quiescence stage of new bone [28,29]. A bone remodeling cycle takes approximately four to eight months [30], and the purpose of constant bone remodeling is to maintain a normal bone mass as a dynamic equilibrium [31,32] to secure the integrity of the skeleton, and these series are regulated by systemic and local factors [30].

Osteoblasts originate from marrow mesenchymal stem cells and produce various extracellular matrix proteins that control bone formation and bone resorption [17]. Mesenchymal progenitors trigger the activation of canonical Wnt/β-catenin signaling to promote the differentiation of osteoblasts, which leads to improved bone strength and the inhibitition of osteoclast differentiation [33]. The runt-related transcription factor 2 (Runx2) is the earliest osteoblast marker for the differentiation of precursor cells in the osteoblast lineage [34]. Alkaline phosphatase (ALP) and type I collagen are secreted by differentiated osteoblasts and are required for the bone-matrix synthesis and subsequent mineralization [35]. In addition, mature osteoblasts produce osteocalcin, osteopontin, and ostenectin to regulate matrix mineralization and the receptor activator of nuclear factor-kappa B ligand (RANKL) to accomplish osteoclast differentiation [30].

Mature osteoclasts are mononuclear precursors of osteoclasts collected to form multinucleated osteoclasts [36]; they contribute to bone resorption and control osteoblast differentiation [37]. At the bone resorption stage, osteoclasts gathering on the surface of the bone secrete acids and enzymes to absorb the bone matrix and release the transforming growth factor-beta (TGF-β) and insulin-like growth factors (IGFs), which regulate the activity of osteoblasts [38]. The ephrin B2 ligand expressed by osteoclasts binds to the EphB4 receptor, which is found on the membrane of osteoblasts, to promote osteoblast differentiation and to suppress osteoclast differentiation [39]. In addition, the secretion of sphingosine 1-phosphate (S1P) by osteoclasts recruits osteoblast progenitor cells to the bone resorption sites and also stimulates these progenitor osteoblasts’ differentiation by stimulating the ephrin B2/EphB4 pathway [30,40]. The main regulator of osteoclast differentiation is the RANKL/receptor activator of nuclear factor-kappa B (RANK)/osteoprotegerin (OPG) pathway, which is based on osteoblasts generating RANKL to bind with the receptor RANK on the surface of the osteoclast precursors. This improves the differentiation and maturation of osteoclasts. Additionally, osteoblasts are also involved in the release of OPG to attenuate the stimulant pathway [41]. Furthermore, the macrophage colony stimulation factor (M-CSF) is another modulator of osteoclast differentiation produced by osteoblasts [41].

Thus, there is a close link between osteoclastic bone resorption and osteoblastic bone formation. Osteoclastic differentiation and bone resorption are regulated by osteoblasts, while osteoblastic differentiation and bone formation are controlled by osteoclasts [37]. In normal bone remodeling, the resorbed bone is entirely replaced by the same amount of new bone at the same location [30]. In addition, at the bone formation stage, the subsequent remodeling of the matrix and the newly synthesized protein matrix form the new bone. As such, bone remodeling plays an important role in maintaining healthy bone mineralization to maintain the hardness and strength of bone [42,43].

### 2.2. The Impairment of Bone Remodeling during CKD

To maintain the integrity of the skeleton, the balance between bone formation and bone resorption must be maintained [44]. The unbalanced regulation of bone remodeling can result in a variety of bone diseases [45]. As CKD progresses, the imbalance leads to different types of pathological bone diseases [46].

**Figure 2:**
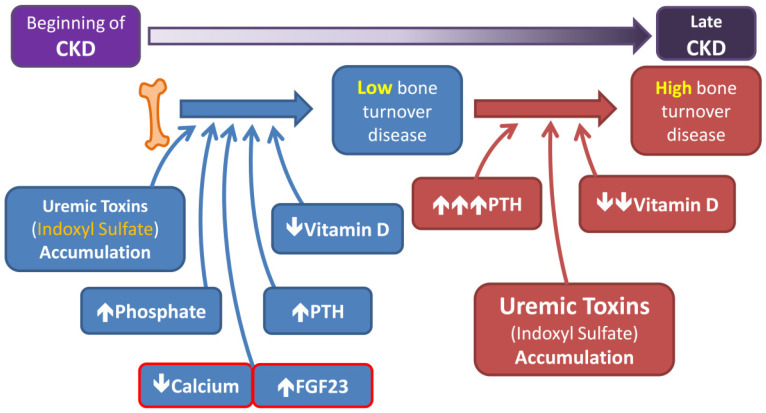
Bone disorders in CKD. When CKD occurs, the accumulation of uremic toxins (indoxyl sulfate) can directly damage bone cells. Early on in CKD, an electrolyte imbalance and decreased vitamin D levels cause low-turnover bone disease despite a slight increase in PTH. However, when the renal function further deteriorates, the serum PTH levels become persistently high and vitamin D levels may decline more, which may cause high-turnover bone disease, as this can override peripheral PTH resistance and other bone-formation inhibitors.

In the early stage of CKD, low-turnover bone disease is common in CKD patients and is characterized by an extremely slow rate of bone formation (Figure 2) [47]. Low-turnover bone diseases include adynamic bone disease, aluminum-induced bone disease, and osteomalacia [48]. In adynamic bone disease, mostly observed in early dialysis patients with the beginnings of bone deterioration, PTH synthesis and secretion decrease, usually due to chronic aluminum intake and high vitamin D intake [49]. Osteomalacia entails a very low rate of bone formation and defective bone mineralization [48]; it is associated with metabolic acidosis and reduced active vitamin D [50]. In contrast, in the end stages of CKD, high levels of PTH cause high-turnover bone diseases, which involve excessive osteoclastic bone resorption and bone marrow fibrosis, such as osteitis fibrosa cystica [51].

As mentioned above, bone disorders in CKD patients occur due to two main reasons: (1) an imbalance of the mineral and hormonal disruption, which reduces bone quantity, and (2) accumulated uremic toxins, which damage bone quality. Fukagawa et al. stated that PTH-stimulated intracellular cyclic adenosine monophosphate (cAMP) production is suppressed by IS, which is associated with the inhibition of PTH receptor expression and causes oxidative stress in the bone cells of CKD patients [52]. They asserted that higher levels of IS could induce adynamic or low-turnover bone disease due to skeletal resistance to PTH through IS [52]. Additionally, the effects of IS on bones include a modified chemical composition, which can involve changes to the mineral/matrix or pentosidine/matrix ratio and increased carbonate substitution [7], which can result in greater bone quality degradation, increasing the hip fracture rates in CKD patients [14]. These conditions affect the quality of life of CKD patients [53] and result in huge costs to healthcare systems [54].

## 3. Aryl hydrocarbon Receptor (AhR) and CKD

### 3.1. AhR Signaling Pathway

AhR is a ligand-activated transcription factor belonging to the Per-Arnt-Sim protein superfamily. In the cytoplasm, AhR is in an inactive form that bonds with several proteins, e.g., Hsp90, ARA9, and p23 [55]. After being exposed and binding to its ligand/agonist, the configuration of AhR transforms and the nuclear transcription site is exposed, which allows AhR to translocate into the nucleus and heterodimerize with the aryl hydrocarbon receptor nuclear transfer protein (ARNT) [56]. In the nucleus, the heterodimer of AhR/ARNT can induce a xenobiotic response element (XRE) sequence to stimulate gene transcription, resulting in various biological effects, such as on toxicity, immune response [57], and bone remodeling [58]. In addition, AhR regulates downstream gene expression, such as cytochrome P450, family 1, member 1A (CYP1A1), CYP1A2, CYP1B1, and the AhR repressor (AhRR) [59,60]. Sometimes, AhR, even without XRE, experiences other transcriptional factor interactions [61].

AhR also controls the metabolism of certain transcription factors and their components. In fact, AhR has been labeled as an E3 ubiquitin protein ligase and can induce the degradation and proteasome-controlled ubiquitination of target proteins. For example, ligand-bound AhR combines with the Cullin 4B (CUL4B)-based E3 ubiquitous ligament to form a CUL4B–AhR complex, whose goal is to degrade the ESR [62]. The activity of the transcription factor or the ubiquitin function of the AhR E3 ligase depends on the availability and function of ARNT. ARNT is an AhR transcription factor that controls the level of AhR; thus, ARNT is reduced or inhibited and AhR E3 ligase activity is enhanced [63]. In addition, the ARNT isolated from AhR stimulates hypoxia-inducible factor 1 subunit alpha (HIF1α) degradation and inhibits HIF1α-driven transcriptional programs, which control T cell function and metabolism [64].

Furthermore, as with the activation of some transcription factors, AhR indirectly regulates NF-ƙB as the suppressor of cytokine signaling 2 (SOCS2)-dependent mechanisms and directly interacts with other components of RelA, RelB, and other NF-ƙB signaling complexes [65,66].

### 3.2. AhR Ligands

AhR possesses numerous exogenous ligands, such as 2,3,7,8-tetrachlorodibenzo-p-dioxin (TCDD) [67], 3-methylcholanthrene (3-MC) [68], and benzo[a]pyrene (BaP) of polycyclic aromatic hydrocarbons (PAHs) [69], as well as various endogenous ligands, such as IS, indole-3-acid-acetic (IAA) [70], KYN [71], and kynurenine’s metabolites: kynurenic acid [72] and quinolinic acid [73]. 

Among the uremic toxins, those derived from tryptophan metabolism are of particular importance because they are associated with cardiovascular toxicity; moreover, they are shown to be potent AhR ligands [74]. For patients with CKD, the essential amino acid tryptophan is metabolized via the gastrointestinal tract into three main groups: (1) indole, (2) KYN, and (3) tryptamine [75]. These are explained separately as follows: (1) Indoles are the intestinal microorganisms which directly transform tryptophan into such molecules as IS, IAA, indole-3-aldehyde, indole-3-acetaldehyde, and indole-3-propionic acid [76,77]. (2) The rate-limiting enzyme indoleamine 2,3-dioxygenase 1 (IDO1) produces KYN and downstream products, such as kynurenic acid and quinolinic acid [78,79]. Wang et al. found that kynurenic acid controlled the level of inflammation [80], and Kalaska et al. pointed out that the accumulation of KYN metabolites in the blood leads to neurological diseases and susceptibility to infectious diseases, anemia, lipid metabolism disorders, and hypertension in CKD [71,81]. (3) Tryptophan is decarboxylated to tryptamine, which is upstream of serotonin and melatonin [82]. 

In summary, diet, host metabolism, and environmental substances offer multiple AhR ligands that are likely to affect the cell growth process and inflammation. The following sections discuss the associations between AhR ligands and bone development in CKD patients.

### 3.3. Activation of AhR through Is Worsens Renal Damage in CKD

CKD patients are exposed to various uremic toxins that are the endogenous ligands of AhR. Lu et al. stated that AhR activation has a pathogenic effect in nephrectomy rats and that there is a positive relationship between renal AhR expression and CKD severity [83]. Moreover, Ichii et al. provided evidence of in vivo and in vitro studies showing IS-activated AhR to induce renal pro-inflammatory phenotypes, podocytes injury, and progressive glomerular damage [84]. Dou et al. found that elevated serum AhR-activating potential was closely related to the IS concentration and the estimated glomerular filtration rate (eGFR) in CKD patients and 5/6 nephrectomy mice [23]. During the analysis of endothelial cells under IS and IAA stimulation, Gondouin et al. proved that the genes of AhR, such as CYP1A1 and CYP1B1, were upregulated [85]. 

In addition, Ng et al. found that IS downregulated the expression of the Mas receptor through the organic anion transporter (OAT) 3/AhR/signal transducer [86]. The binding of IS with the AhR complex enhances oxidative stress, inflammation, and the synthesis of the renin-angiotensin system (RAS) proteins to ultimately promote renal fibrosis [87]. Thereby, upregulating the transforming growth factor-beta 1 (TGF- β1) in proximal renal tubular cells aggravates CKD [86]. Lu et al. demonstrated that IS activated AhR to contribute to renal tubulotoxicity via upregulating arachidonate 12-lipoxygenase (ALOX12) with the endovanilloid 12(S)-hydroxyeicosatetraenoic acid (12(S)-HETE) synthesis pathway to induce the transient receptor potential vanilloid 1 (TRPV1) hyperfunction [88]. Lee et al. applied AhR-knockout and pharmacological-inhibitor α-naphthoflavone models to explore the role of AhR in diabetic nephropathy. They discovered that the kidneys of diabetic mice presented signs of oxidative stress, such as extracellular matrix accumulation, macrophage infiltration, and mesangial cell activation because of the elevation of AhR. Therefore, uremic toxins/the AhR signaling pathway result in harmful effects, most of which are related to CKD and have an impact on the cardiovascular system [89]. Therefore, the IS/AhR pathways affect renal function as a vicious circle in CKD patients.

### 3.4. Oxidative Stress Accentuated by the IS/AhR Pathway

It is well known that IS enhances the response of oxidative stress and induces the production of ROS through the activation of nicotinamide adenine dinucleotide phosphate (NADPH) oxidases [90]. Sun et al. stated that a high level of serum IS enhances the production of free radicals, promotes oxidative stress, and induces inflammatory gene expression in the kidneys [91].

Several studies showed that IS promoted the damage of renal tubulointerstitial cells in CKD patients by inducing oxidative stress and activating the NF-ƙB pathway, which produced various cytokines and inflammatory mediators to enhance kidney damage [92]. Furthermore, in the study of Stockler-Pinto et al., they stated that the IS induced monocyte-mediated inflammation and adipocytes to secrete tumor necrosis factor-α (TNF-α) and interleukin (IL)-6 by oxidative stress in CKD patients [93]. In fact, Borges et al. discovered that CKD patients had a potential possibility of developing cardiovascular disease; one of the reasons for this was an increased expression of IL-6 and monocyte chemoattractant protein-1 (MCP-1) connected to higher IS and IAA [94]. Additionally, in renal bone disorders, Kim et al. pointed out that IS acted as a bone toxin to induce oxidative stress in primary osteoblast cell cultures [95]. The accumulation of uremic toxins in CKD is correlated with complications, such as cardiovascular disease, muscle wasting, renal anemia, and CKD–MBD [96,97,98,99]. These studies assumed that IS-induced oxidative stress facilitated a variety of mechanisms of CKD-related complications.

## 4. The Impairment of Bone Metabolism through the IS/AhR Pathway

Recent documents have shown that AhR has brought more scientific attention to the effects of bone remodeling [24,25]. As we know, AhR is expressed in bone cells, including osteoblasts and osteoclasts [100]. We explore the role of AhR and its associated signaling pathways in bone remodeling in order to provide a new treatment concept.

### 4.1. Activated AhR Destroys Bone Remodeling

AhR ligands can suppress osteoblast differentiation, as demonstrated in many toxicological studies [101,102]. Nguyen et al. confirmed that activated AhR by TCDD blocked the differentiation of osteoblasts from bone-marrow-derived stem cells [103]. Herlin et al. described that TCDD, via the AhR pathway, might cause thin cortical bones, a hard bone matrix, and mechanically weak bones and might raise trabecular bone volume fraction [58]. TCDD changes the structure of the AhR transcription activation domain to disturb bone remodeling and to decrease bone strength [104]. Moreover, AhR antagonists, such as resveratrol (RSV), increased the bone mineral density (BMD) and bone mass in mice, in the study by Yu et al. [105]. Further, Kharouf et al. stated that RSV enhanced the activity of osteoblasts and decreased the activity of osteoclasts in the treatment of orthodontics [106]. In other studies, Jameel Iqbala et al. demonstrated that the smoke toxins, BaP and TCDD, interacted with AhR to induce osteoclastic bone resorption through the activation of CYP1 families [107]. However, Voronov et al. reported that BaP directly suppressed the differentiation and function of osteoclasts, which were dependent on the AhR–RANKL pathway [108].

It is worth mentioning that KYN inhibits the proliferation and differentiation of osteoblasts through the stimulation of AhR via the activation of the ERK signaling pathway in a collagen-induced arthritis mouse model [24,71]. At the same time, KYN promoted the expression of the AhR target gene CYP1A1 in osteoclasts and enhanced the osteoclast activity and exacerbation of the bone resorption issue [109]. These various research works illustrate that the different ligands bound with AhR play diverse roles. The reasons may depend on different capacities and concentrations of ligands or the duration of the ligand stimulation.

### 4.2. The IS/AhR Pathway Impairs Osteoclastogenesis 

In an in vivo study, Mozar et al. stated that IS blocked the differentiation/function of osteoclasts and the activity of bone resorption in a dose-dependent manner after being cultured for five days with more than 200 μM of IS with 3 mM of NaH_2_PO_4_ salt [110]. Their results suggested that osteoclast and osteoblast function was inhibited by IS through the mitogen-activated protein kinase (MAPK) ERK1/2, p38, JNK, and Akt pathways, which result in bone remodeling being destroyed in patients with CKD [110]. Additionally, 30–300 μM of IS inhibited the development of Raw 264.7 cells, osteoclast precursors, and bone-marrow-derived macrophages, as well as blocking RANKL-induced differentiation into mature osteoclasts after culturing for five days, according to the study by Watanabe et al. [19].

**Figure 3:**
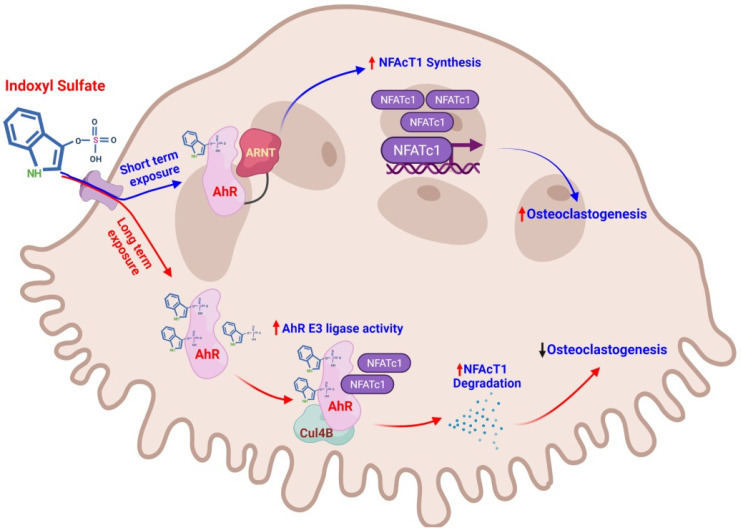
Time and dose dependent of IS on osteoclastogenesis. Short-term exposure in a low IS dose and ARNT are available, and AhR works as a ligand-activated transcription factor, increasing NFATc1 expression, and thus increasing osteoclastogenesis. On the contrary, long-term exposure in high doses of IS and ARNT are inaccessible, and AhR functions as an E3 ubiquitin ligase, leading to the proteasomic degradation of NFATc1 and thereby inhibiting osteoclastogenesis.

In the differentiation stage of osteoclast precursors, the nuclear factor of activated cytoplasmic T lymphocytes 1 (NFATc1) plays a dominant role [111]. However, NFATc1 is not only a significant transcription factor during the genesis of osteoclasts [112] but is also an AhR target protein that could be influenced by IS. In our previous study, we found that IS, through AhR signaling in dose- and time-dependent manners, affected NFATc1 and osteoclastogenesis. Through the IS/AhR/NFATc1 pathway in the Raw 264.7 cell line, low concentrations (less than 100 μM) and short durations (three days) of IS stimulation enhance osteoclastogenesis; in contrast, high concentrations (higher than 500 μM) and long durations (five days) of IS stimulation suppress osteoclastogenesis [113]. Therefore, the effects of IS on osteoclastogenesis can cause promotion or inhibition (Figure 3).

As stated by Luecke-Johansson et al., the E3 ubiquitin ligase function competed with the AHR transcription factor function, which depends on the ARNT. Higher ARNT levels promote AhR/ARNT combinations, while lower ARNT levels enhance AhR E3 ligase activity [63]. Our study also demonstrated the different levels and durations of IS stimulation, switching the role of AhR from a ligand-activated transcription factor to an E3 ubiquitin ligase; ARNT may be a key factor in the regulation of these dual functions of AhR under IS treatment [113] (Figure 2).

As there is still no effective treatment to improve osteoclast functioning in most CKD patients, the IS/AhR/NFATc1 pathway could demonstrate that excessive uremic toxin elimination may correct abnormal osteoclast development. In addition, AhR antagonists can serve as new drugs against renal osteodystrophy to prevent the deterioration of bone metabolism associated with CKD.

### 4.3. The IS/AhR Pathway Damages Osteoblastogenesis

The first study describing IS accumulation in serum in connection with the suppression of osteoblast function was by Fukagawa et al. in 2006 [114,115]. They found that IS induced skeletal resistance to PTH in cultured osteoblast cells and that the production of free radicals from osteoblasts was positively correlated with the concentration of IS [52]. Watanabe et al. continued to study the direct effect of IS on bone turnover in adult rats with parathyroidectomy (PTX). As PTX decreased bone turnover, they found that IS further exacerbated low bone renewal by inhibiting bone formation, even without PTH [116].

**Figure 4:**
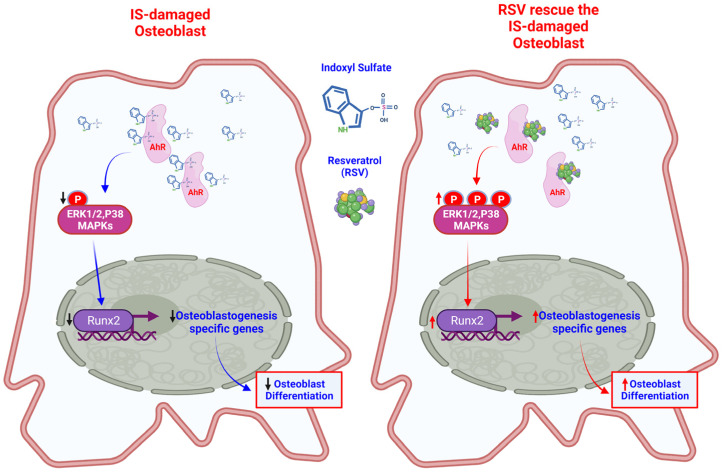
Resveratrol (RSV) restores the reduction in ERK and p38 MAPK phosphorylation caused by IS in osteoblasts. IS inhibits the phosphorylation of ERK and p38 MAPK through the AhR pathway in the osteoblast cytoplasm, which then reduces the expression of Runx2 to impede osteoblast differentiation. RSV, an AhR antagonist, restores the reduction in ERK and p38 MAPK phosphorylation due to IS and then improves the expression of Runx2 to promote osteoblast differentiation.

Several research works have explored the effects of IS on different maturation stages of osteoblasts based on various markers, such as ALP, type I collagen, Osterix and Runx2 in early stage of osteoblast differentiation [117], and osteocalcin (OCN), OPG, bone morphogenetic protein 2 (BMP2), and RANKL in the late stage [118] [19,95,119]. In particular, Runx2 is an important transcribing factor for osteoblast differentiation and is a crucial regulator of bone formation [120]. There are also some studies of the AhR signaling pathway and multiple transductions to detect cell development in a uremic scenario, especially the MAPK pathway [121]. An understanding of the MAPK pathway in osteoblasts is important in order to recognize the physiological control of bone formation [122]. There have been many in vivo and in vitro studies on this topic [123]. In our recent study, we proved that the phosphorylation of ERK and p38 MAPK through the AhR pathway could be inhibited by IS in osteoblasts, which then reduced the expression of Runx2 to impede osteoblast differentiation [119]. This showed that damaged osteoblasts and damage to bone texture can be obtained through IS/AhR/ERK and p38 MAPK/Runx2 [119]. In addition, Yu et al. showed that TCDD also stimulated AhR to inhibit osteoblast development through the signaling pathway of ERK/MAPK [24] (Figure 4).

Recently, a number of reports have suggested that IS itself could be a bone toxin [110]. In the study of Kim et al., they believed IS directly inhibited osteoblast differentiation and induced osteoblast apoptosis through the caspase activity, which is mediated by IS-induced free radical production to cause apoptosis [95].

### 4.4. The AhR Antagonist Ameliorates IS-Induced Worsening of Bone Remodeling

Similar to AhR agonists/ligands, there are also several AhR antagonists [61], such as RSV (3,5,49-trihydroxystilbene) [124] and 6,2ʹ,4ʹ,-trimethoxyflavone [125], which can reverse the effect of AhR ligands. RSV, the most discussed antagonist, is a molecule found in red wine that blocks the induction of CYP1A1 by preventing the binding of AhR to promoter sequences [126]. In our recent study of osteoblasts, RSV was shown to restore the reduction in ERK and p38 MAPK phosphorylation caused by IS and then improve the expression of Runx2 to promote osteoblast differentiation [119] (Figure 3). In another in vitro study on osteoblasts, Dai et al. proved that RSV enhanced the proliferation of human mesenchymal stem cells (MSCs) and the differentiation of osteoblasts in a time- and dose-dependent manner through ERK1/2 activation, thereby increasing the activity of alkaline phosphatase and calcium deposited in human MSC cultures [127].

In their studies on osteoclastic cells, Naruse et al. [128] and Voronov et al. [108] stated that RSV did not control osteoclastogenesis but could lift the blockage of osteoclastic resorption induced by AhR antagonists. In contrast, He et al. found that RSV decreased RANKL-induced osteoclast differentiation and increased differentiated osteoclast apoptosis at non-toxic, dose-dependent concentrations by suppressing RANKL-induced ROS generation [129].

## 5. Conclusions

In patients with CKD, the accumulation of uremic toxins, such as IS and KYN, can increase the overexpression of AhR, thus inducing harmful signaling and triggering the deterioration of bone remodeling. In fact, bone remodeling is a continuous process and a dynamic equilibrium between osteoblasts’ bone formation and osteoclasts’ bone resorption. Though the different ligands of AhR have inconstant effects on bone remodeling, it has been verified that AhR plays an important role in bone formation and bone resorption and is the key signal pathway of bone remodeling. Thus, the correction of the production of oxidative stress and inflammatory cells triggered by IS provides potential therapeutic options against the many complications of CKD. 

The uncertainty of clinical application merits further investigation; particularly with respect to the effects of high and low IS concentrations on osteoclasts and osteoblasts. There are various impacts and responses from sequential stages of CKD with different IS levels. Additionally, different levels of PTH can also affect the toxic effects of IS on bones. Therefore, in clinical practice, there should be more experiments designed to achieve the safest and most appropriate level of IS in patients with CKD. 

## Data Availability

This is a narrative review article. The primary collection of documents for analysis and review comes from PubMed.

## Abbreviations

AchE	Acetylcholinesterase
AhR	Aryl Hydrocarbon Receptor
AhRR	AhR Repressor
ALOX12	Arachidonate 12-Lipoxygenase
ALP	Alkaline Phosphatase
AP-1	Activator Protein 1
ARNT	Aromatic hydrocarbon Receptor Nuclear Transfer Protein
BaP	Benzo[a]pyrene
BMD	Bone Mineral Density
BMU	Basic Multicellular Unit
cAMP	cyclic Adenosine Monophosphate
CKD-MBD	Chronic Kidney Disease-Mineral and Bone Disorder
CTLs	Cytotoxic T Lymphocytes
CUL4B	Cullin 4B
CYP1A1	Cytochrome P450, family 1, member 1A
DCs	Dendritic Cells
eGFR	estimated Glomerular Filtration Rate
EPO	Erythropoietin
FGF23	Fibroblast Growth Factor 23
HIF1α	Hypoxia Inducible Factor 1 Subunit Alpha
HUVECs	Human Umbilical Vein Endothelial Cells
IAA	Indole-3-Acid-Acetic
IDO1	Indoleamine 2,3-Dioxygenase 1
IGFs	Insulin-like Growth Factors
iPTH	intact PTH
IS	Indoxyl Sulfate
KYN	Kynurenine
MAPK	Mitogen-activated Protein Kinase
3-MC	3-Methylcholanthrene
M-CSF	Macrophage Colony Stimulation Factor
MSCs	Mesenchymal Stem Cells
NADPH	Nicotinamide Adenine Dinucleotide Phosphate
NFATc1	Nuclear Factor of Activated T-cells, Cytoplasmic 1
NF-ƙB	Nuclear Factor-kappa B
OAT	Organic Anion Transporter
OPG	Osteoprotegerin
PAHs	Polycyclic Aromatic Hydrocarbons
PTH	Parathyroid Hormone
PTX	Parathyroidectomy
RANK	Receptor Activator of Nuclear factor-kappa B
RANKL	Receptor Activator of Nuclear factor-kappa B Ligand
RAS	Renin-Angiotensin System
ROS	Reactive Oxygen Species
RSV	Resveratrol
Runx2	Runt-related Transcription Factor 2
S1P	Sphingosine 1-Phosphate
SOCS2	Suppressor of Cytokine Signaling 2
STAT3	Signal Transducer and Activator of Transcription 3
12(*S*)-HETE	12(*S*)-Hydroxyeicosatetraenoic Acid
TCDD	2,3,7,8-Tetrachlorodibenzo-p-Dioxin
TGF-β	Transforming Growth Factor-beta
Th17	T helper 17
TRPV1	Transient Receptor Potential Vanilloid 1
XRE	Xenobiotic Response Element
